# *Bartonella henselae* DNA in Seronegative Patients with Cat-Scratch Disease

**DOI:** 10.3201/eid2405.152033

**Published:** 2018-05

**Authors:** Masashi Yanagihara, Hidehiro Tsuneoka, Ayano Tanimoto, Ken-ichiro Otsuyama, Jun Nishikawa, Tomohiro Matsui, Junzo Nojima, Kiyoshi Ichihara

**Affiliations:** Yamaguchi University, Ube, Japan

**Keywords:** Cat-scratch disease, *Bartonella henselae*, peripheral blood, real-time PCR, indirect fluorescence antibody assay, Japan, zoonoses, bacteria

## Abstract

We used real-time PCR to detect *Bartonella henselae* DNA in 7.9% (5/63) of blood specimens from seronegative patients in Japan suspected of having cat-scratch disease. The combined use of serologic tests and real-time PCR to analyze blood specimens is recommended for the prompt, noninvasive laboratory diagnosis of cat-scratch disease.

Cat-scratch disease (CSD) is a worldwide zoonosis caused by *Bartonella henselae* (*1*). Its clinical manifestations vary from typical CSD with regional lymphadenopathy to atypical or systemic CSD, including prolonged fever without lymphadenopathy. Because isolation of *B. henselae* by culture is difficult (*2*), detection of *B. henselae* DNA in lymph nodes by PCR and serologic testing using indirect fluorescence antibody (IFA) assay is widely used for laboratory diagnosis of CSD (*2*–*4*). Isolation of *B. henselae* DNA from blood specimens of immunocompetent CSD patients has been sporadically described, suggesting that it might be useful, especially for cases in which lymphadenectomy or biopsy is not feasible or serologic results are equivocal (*5*–*9*). However, the usefulness of serologic testing, coupled with detection of *B. henselae* DNA from blood specimens, is not well described. We report the clinical utility of the combined use of IFA and real-time PCR to analyze blood specimens for noninvasive screening of CSD.

During April 2009–May 2014, eighty immunocompetent patients (73 children, 7 adults) in Japan who were suspected of having CSD because of fever with or without lymphadenopathy, and a history of contact with cats or dogs were referred to us for serologic and molecular diagnosis of CSD. We conducted serologic testing using IFA (*3*,*4*); diagnosis was based on elevated titers of IgM (>20) or IgG (>256). The sensitivity and specificity of our IFA were 69% and 100%, respectively (*4*). Real-time PCR (rPCR) detected specific *B. henselae virB4* DNA from blood specimens as reported previously (*9*). In brief, we extracted DNA from peripheral blood using a QIAamp DNA Mini Kit (QIAGEN, Hilden, Germany). The 20-μL PCR mixture contained 10 μL 2× LightCycler 480 Probes Master Mix (Roche Diagnostics, Mannheim, Germany), 0.4 μmol/L of each primer (forward: 5′-AGCGAAGAAAACACAATCTGAA-3′; reverse: 5′-TCCATAGCTTTCCAATCCTTCT-3′), 0.1 μmol/L Universal ProbeLibrary probe no. 135 (Roche Diagnostics), and 5 μL DNA. We conducted the reaction using a LightCycler 480 instrument (Roche Diagnostics) under the following conditions: denaturation at 95°C for 5 min and 45 cycles of 95°C for 10 s, 60°C for 30 s, and 72°C for 1 s. We determined crossing point (Cp) values using the second derivative maximum method and analyzed all samples in triplicate. 

This assay detected all 56 specimens evaluated as *B. henselae* (*10*), but other *Bartonella* species were not identified (*9*). The lower quantitative detection limit of this assay was 4.6 DNA copies/reaction using serial dilutions of plasmid DNA.

Of the 80 patients with suspected CSD, 17 (21.3%) were serologically positive for *B. henselae* by IFA. *B. henselae* DNA was amplified from the peripheral blood of 11 (13.8%) patients by rPCR. Six patients were positive by both IFA and rPCR. CSD was diagnosed in 22 (27.5%) of the 80 patients (Table). Cp values of the IFA-positive and -negative patients did not differ.

**Table:** Description of patients with cat-scratch disease and results of IFA and rPCR of blood specimens, Japan, April 2009–May 2014*

Patient no.	Patient age, y/sex	Fever	Regional lymphadenopathy	History of contact with animal	IFA titer		Cp of rPCR, mean ± SD	Complication
IgM	IgG
1	15/M	+	+	Cat, dog	40	256	NA	
2	4/F	+	–	Cat	20	512	36.82 ± 0.57	FUO
3	6/F	+	–	Dog	<10	64	33.93 ± 0.39	FUO
4	13/M	+	+	Cat	<10	256	35.98 ± 0.69	
5	14/F	+	+	Cat, dog	<10	256	34.29 ± 0.43	
6	8/M	+	+	Dog	<10	<64	35.35 ± 0.33	
7	10/M	+	+	Cat	20	256	NA	
8	8/F	+	+	Dog	<10	<64	37.18 ± 0.36	
9	10/M	+	+	Cat	20	256	NA	
10	7/F	+	+	Cat	10	<64	35.64 ± 0.27	
11	2/M	+	+	Cat	40	256	NA	
12	15/M	+	–	Cat, dog	>80	512	NA	Neuroretinitis
13	6/F	+	+	Cat	<10	256	37.29 ± 0.15	
14	10/M	+	+	Cat	<10	256	NA	
15	12/F	+	+	Cat, dog	<10	512	33.55 ± 0.33	
16	13/M	+	–	Cat	<10	512	NA	FUO
17	9/F	+	+	Cat	<10	<64	34.84 ± 0.47	
18	4/M	+	+	Cat	20	512	NA	
19	14/F	+	–	Dog, rabbit	80	512	NA	Neuroretinitis
20	56/F	+	–	Cat	<10	256	NA	Neuroretinitis
21	35/F	+	+	Cat	40	1,024	37.19 ± 0.59	
22	7/M	+	+	Cat, dog	<10	512	NA	

*Blank cells indicate no complications. Cp, crossing point value; FUO, fever of unknown origin; IFA, indirect fluorescent antibody test; NA, no amplification by rPCR; rPCR, real-time PCR; +, positive; –, negative.

Despite the high specificity, IFA lacks sensitivity (*4*). This study showed that CSD detection sensitivity increased from 21.3% (17/80) using IFA alone to 27.5% (22/80) with combined use of IFA and rPCR on blood specimens. We attribute this increase to the detection of *B. henselae* DNA in 5 patients with seronegative results. Four patients (nos. 6, 8, 10, and 17) exhibited typical CSD with fever and regional lymphadenopathy, and 1 (no. 3) had fever of unknown origin without lymphadenopathy (Table). After laboratory diagnosis, these patients were treated with macrolides to reduce fever. These observations suggested that these patients were in the initial stages of the illness (before a significant rise of antibodies to *B. henselae*) or that the patients’ immune responses were insufficient to produce antibodies to *B. henselae* (*5*).

We detected *B. henselae* DNA in blood specimens of 35.3% (6/17) of the seropositive patients in our study, whereas previous studies detected DNA in 19.2% (5/26) of blood specimens from seropositive patients (*5*) and 16.7% (3/18) of serum specimens from patients proven to have CSD (*6*). rPCR using blood specimens was not sensitive enough when used alone because *B. henselae* DNA is not present in the bloodstream in all patients. The time points at which *B. henselae* DNA can be detected in blood specimens are still unknown. A previous report described this time point as 3 weeks after onset of lymphadenopathy (*7*), whereas another study considered it to be 3–4 months after infection (*8*). Most specimens used in our study were collected within 3 weeks after symptom onset. Regardless of the days after onset, rPCR testing of blood specimens should be performed actively because patients may experience bacteremia or the shedding of bacterial breakdown products into the bloodstream.

In conclusion, our study showed that rPCR testing of blood specimens can detect *B. henselae* DNA in patients with seronegative results. The combined use of IFA and rPCR on blood specimens is useful for the noninvasive screening of CSD.
